# Mannose-Binding Lectin Promoter Polymorphisms and Gene Variants in Pulmonary Tuberculosis Patients from Cantabria (Northern Spain)

**DOI:** 10.1155/2012/469128

**Published:** 2012-12-04

**Authors:** J.-Gonzalo Ocejo-Vinyals, Lucía Lavín-Alconero, Pablo Sánchez-Velasco, M.-Ángeles Guerrero-Alonso, Fernando Ausín, M.-Carmen Fariñas, Francisco Leyva-Cobián

**Affiliations:** ^1^Servicio de Inmunología, Hospital Universitario Marqués de Valdecilla, Avenida de Valdecilla s/n, 39008 Santander, Spain; ^2^Unidad de Enfermedades Infecciosas, Departamento de Medicina Interna, Hospital Universitario Marqués de Valdecilla, Universidad de Cantabria, 39011 Santander, Spain

## Abstract

Mannose-binding lectin is a central molecule of the innate immune system. Mannose-binding lectin 2 promoter polymorphisms and structural variants have been associated with susceptibility to tuberculosis. However, contradictory results among different populations have been reported, resulting in no convincing evidence of association between mannose-binding lectin 2 and susceptibility to tuberculosis. For this reason, we conducted a study in a well genetically conserved Spanish population in order to shed light on this controversial association. We analysed the six promoter and structural mannose-binding lectin 2 gene variants in 107 patients with pulmonary tuberculosis and 441 healthy controls. Only D variant and HYPD haplotype were significantly more frequents in controls which would indicate that this allele could confer protection against pulmonary tuberculosis, but this difference disappeared after statistical correction. Neither the rest of alleles nor the haplotypes were significantly associated with the disease. These results would indicate that mannose-binding lectin promoter polymorphisms and gene variants would not be associated with an increased risk to pulmonary tuberculosis. Despite the slight trend of the D allele and HYPD haplotype in conferring protection against pulmonary tuberculosis, susceptibility to this disease would probably be due to other genetic factors, at least in our population.

## 1. Introduction

Tuberculosis (TB) is one of the world's most important infectious causes of death worldwide. More than 90 million TB patients were reported to WHO between 1980 and 2005, most of them in Asia and sub-Saharan Africa [1]. Spain is one of the European countries with the highest rates of incidence and prevalence of TB [2].

Approximately 90%–95% of individuals infected with *Mycobacterium tuberculosis* (MTB) are able to mount an immune response that halts the progression from latent TB infection to active TB disease. This is one of the main reasons that would indicate the need to identify and treat all those with risk factors for TB disease [3, 4].

Susceptibility to TB seems to be multifactorial, and the development of active disease would probably be the result of a complex interaction between the host and pathogen influenced by environmental and genetic factors. Numerous host genes are likely to be involved in this process [5–7]. 

Mannose-binding lectin (MBL) is an acute phase protein primarily produced by the liver. One of its main roles is to activate the complement system suggesting that it is one of the most important constituents of the innate immune system [8–12]. The gene encoding MBL has been associated with susceptibility to TB and other infectious diseases [8, 13]. The first mutation in *MBL2*, the gene encoding MBL was found in 1991 [14]. Three structural mutations, affecting codons 52, 54, and 57, in the first exon of the *MBL2* gene (MBL1 is a pseudogene) have been found, and the corresponding alleles were designated D, B, and C, respectively (A is the wild-type allele for all three positions). Moreover, three polymorphisms have also been identified in the *MBL2 *promoter and 5′-untranslated regions: H/L at position −550, X/Y at position −221, and P/Q at position +4 [15].

The effect of the three structural mutations in the *MBL2* first exon involves the impairment of MBL multimerization. This caused decreasing ligand binding and, consequently, a lack of complement activation [16]. In general, all these genetic variants result in a phenotype of low serum MBL levels, which influences the susceptibility to TB and the course of different diseases [13, 17, 18].

To date, controversial results have been reported regarding the relationship between structural genetic variants or polymorphisms of the *MBL2* gene and an increased risk of TB in different populations [19–30]. Several studies have found a significant association between the frequency of structural alleles or promoter polymorphisms and serum MBL levels with susceptibility to TB [20, 23, 24, 26–30] while others did not find any significant association [19, 21, 25]. Recently a meta-analysis of 17 human trials considering the effect of *MBL2* genotype and/or MBL levels and TB infection did not found significant association between *MBL2* genotype and pulmonary TB infection [22]. The majority of studies analysed did not report neither the *MBL2* haplotype nor the promoter polymorphisms. The aim of our study was to analyse if gene variants, promoter polymorphisms, haplotypes, or diplotypes could contribute to increase the risk of active pulmonary TB (PTB) in a human immunodeficiency virus negative genetically homogeneous population (Cantabria, Northern Spain), containing newly diagnosed patients with active disease.

## 2. Material and Methods

### 2.1. Study Population

To investigate the possible association between *MBL2* polymorphisms and PTB infection in our population, we recruited a total of 107 active PTB patients and 441 randomly selected healthy blood donors from Cantabria (northern Spain). All of them were HIV negative. 

The study was conducted at a 1,200-bed community and teaching hospital. Both, blood donors (mean age, 48 years; range, 18–65 years; male/female ratio, 1.3) and PTB patients (mean age, 56 years; range, 23–76 years; male/female ratio, 1.5) were of Caucasian background. The PTB patients group was selected from patients admitted to the Infectious Unit and the Department of Respiratory Medicine (Hospital Universitario Marqués de Valdecilla) from 2008 to 2011 and who fulfilled clinical, radiological, and bacteriological criteria of active PTB according the standards for the diagnosis and classification of TB developed by the American Thoracic Society and the Centers for Disease Control and Prevention (http://www.cdc.gov/mmwr/). Diagnosis of PTB was made clinically and by X-rays and confirmed by bacteriological (microscopy and culture) procedures. We excluded patients with extrapulmonary TB due to dissemination and subsequent involvement of single or multiple nonpulmonary sites. In the same way, we excluded patients with autoimmune or neoplastic diseases, chronic renal failure, transplant individuals, and patients suffering from alcoholism or drug abuse. Controls had neither previous history of TB nor contact with infected patients. Furthermore, we ruled out the presence of active or latent TB in the control group by performing an interferon-gamma release assay (Quantiferon TB Gold, Cellestis Ltd., Carnegie, Victoria, Australia). All the procedures used in the study conformed to the principles outlined in the Declaration of Helsinki. Informed consent was obtained and data anonymously recorded. The study protocol was accepted and approved by the Research Ethics Board of the Hospital.

### 2.2. DNA Extraction and Amplification of Genomic DNA for **MBL2 ** Genotyping

Blood was collected in EDTA-stabilized tubes in compliance with approved protocols from our institution. Genomic DNA from patients and controls was extracted from peripheral blood by using the Maxwell 16 Genomic DNA Purification system. For *MBL2* gene amplification and genotyping, we used the INNO-LiPA *MBL2* (Innogenetics Diagnóstica Iberia S.L.U, Barcelona, Spain), following the manufacturer's instructions. The INNO-LIPA *MBL2* is a line probe assay, designed for genotyping the 6 variations in the human *MBL2* gene (−550G > C, −221G > C, +4C > T, R52C, G54D, and G57E) which leads to analyse the seven common haplotypes and the 28 possible resulting diplotypes.

## 3. Statistical Analysis

Frequencies of alleles and diplotypes of patients and healthy controls were estimated by direct counting. Alleles and genotypic (dyplotypes) frequencies were compared by *χ* ^2^ test or the Fisher's exact test when necessary. *P* values with Yates correction and odds ratio (OR) with 95% confidence intervals (CI) were calculated using SPSS version 12.0 (SPSS Inc, Chicago, IL, USA). *P* < 0.05 was considered statistically significant. Hardy-Weinberg equilibrium (HWE) was tested in patients and controls for all the analysed parameters. Bonferroni correction for multiple comparisons was applied in order to avoid false positive results. 

For haplotype analysis, frequencies and linkage disequilibrium were calculated trough the expectation maximization algorithm using the SNPStats web-based tool (http://bioinfo.iconcologia.net/SNPstats/). To determine the linkage disequilibrium between pairs of alleles, we calculated the D′ statistic. Comparisons between patients and controls were performed by *χ* ^2^ test or the Fisher's exact test considering each haplotype like an allele.

Statistical power was calculated by using the PS power and sample size calculation software version 3.0, (http://biostat.mc.vanderbilt.edu/PowerSampleSize).

## 4. Results and Discussion

### 4.1. *MBL2* Structural Variants, Promoter Polymorphisms, and Genotypes

Frequencies of *MBL2* alleles are shown in Table 1. All the structural genetic variants and the promoter polymorphisms were within the range of HWE. The average heterozygosity in the control group was 0.88 when the seven alleles of the *MBL2* gene were analysed, being this frequency higher than others previously reported in different populations [30].

There was no significant difference in the frequencies of the different promoter polymorphisms. Regarding the structural variants, only D allele was significantly more frequent in controls (*P* = 0.014, OR 0.33, and CI 95% 0.13–0.84), but this significance disappeared after Bonferroni correction. 

Table 2 shows the frequency of the *MBL2* haplotypes in controls and PTB patients from Cantabria compared with other previously reported populations. As with D structural variant, HYPD showed the same significant difference between PTB patients and controls which disappeared after Bonferroni correction. All the promoter polymorphisms and structural variants conforming the different haplotypes were in linkage disequilibrium (*P* < 2*e* − 16).

### 4.2. *MBL2* Complete Genotypes (Diplotypes) in PTB Patients and Healthy Controls

The frequencies of all the possible combinations of the seven haplotypes that appeared in both groups are shown in Table 3. No significant differences were found in any of the complete diplotypes between PTB patients and healthy controls.

All seven haplotypes were present in both groups following the same order of frequency in them, giving rise to 22 different *MBL2* diplotypes in our subjects. Eighteen of these diplotypes were present in both groups, 5 in either the PTB patients or the control group, and six diplotypes were not observed in any of the groups.

HYPA was the most frequent haplotype, followed by LYQA, LXPA, LYPB, LYPA, HYPD, and LYQC, respectively. When we regrouped the 22 observed diplotypes in all the possible genotypic combinations (structural-structural, structural-polymorphism, and polymorphism-polymorphism), we did not find any significant difference (data not shown).

## 5. Discussion

Innate immunity is one of the most important barriers against invading pathogens. The complement system gets activated when these microorganisms are detected, resulting in biochemical pathways that lead to the destruction of the infectious agent. One of these pathways that make up the complement system is the lectin pathway in which MBL plays the main role. MBL protein is therefore important, especially in first-line defense against invading pathogens. It has been reported that low levels of circulating MBL may predispose against infectious diseases [8, 31, 32].

Structural variants are found at the coding regions of the *MBL2* gene that lead to low or near absent serum MBL levels in heterozygosis and homozygosis, respectively. Low-serum levels of MBL are associated with defects in opsonization, resulting in recurrent infections mainly during infancy [31]. Due to a strong linkage disequilibrium between the polymorphisms present in the promotor and the structural variants in exon 1 of the human *MBL2* gene, only seven common haplotypes have been described (HYPA, LYPA, LXPA, LYQA, HYPD, LYPB, and LYQC) which give rise to 28 possible haplotype combinations. The frequencies of the seven haplotypes vary considerably between populations [17, 18]. Among haplotypes carrying the wild-type A allele, HYPA results in the production of higher amounts of MBL, whereas LXPA is associated with lower serum MBL levels.

Several groups have studied *MBL2* genetic variants and PTB, suggesting a partial protective effect of heterozygosity for *MBL2 *variant alleles against PTB [33–35], whereas others have pointed toward a susceptibility to PTB for homozygous carriers of *MBL2* variant alleles [36].

Previous studies have found controversial results, at least at a genetic level. Some authors have reported a lower frequency of allele B in Afro-Americans, but not in Caucasian or in the so-called “Hispanic” TB patients [28]. However, other reports have not found any significant differences in the frequency of structural *MBL2 *alleles between PTB Caucasian patients and control subjects. Nevertheless, when they included the promoter polymorphisms according to high serum MBL levels (*YA/YA*, *YA/XA*, *XA/XA*, and *YA/O*), low MBL levels (*XA/O*), and deficient MBL individuals (*O/O*),a significant difference in diplotype frequency was revealed [25, 27, 29]. Finally, another study has found a significantly increased frequency of *O/O* diplotype of structural polymorphism and of *Y/Y* diplotype of promoter polymorphisms in HIV-TB+ patients compared with controls [24].

In the present study, we have investigated whether structural variations or promoter polymorphisms in the *MBL2* gene considering them individually of regrouped might be associated with PTB in Northern Spain. Our results after statistical correction show that there is no differences neither in the frequencies of polymorphisms in the promoter and 5′-untranslated region nor in the structural variants of the exon 1 of the *MBL2* gene between PTB patients and control subjects when we considered them individually. 

Lack of concordance of our results and those from other studies, specifically another Spanish report that studied *MBL2* gene variants in the population from Canary Islands, [27] could be explained, at least, by the genetic characteristics of both pathogens and hosts. There are, consequently, two possibilities to understand these differences among insular and peninsular Spaniards.

First of all, it should be considered the different ethnic background of geographically apart populations [37–40]. Genetic studies have demonstrated that an aboriginal African background still persists in inhabitants from Canary Islands. Estimates of genetic contribution to the Canary Islanders from their putative parental populations based on mtDNA and other genetic markers are 43% Berbers, 35% Peninsular Spaniards, and 21% Guineans (being the Spanish nuclear contribution due to males and practically all the Berber and Guinean due to females). On the other hand, Cantabrians, at the North of the Iberian Peninsula, appear as a semi-isolated result of an ancient indigenous substrate more or less mixed with more recent immigrants. This population seems to be a genetically well-differentiated community, as deduced from uniparental and autosomal markers, perhaps to a higher level than their neighbours, the Basques, the most reputed European isolate on linguistic grounds [41–43].

Secondly, another explanation could be due to the genetic background within *M. tuberculosis* because variability among different strains of *M. tuberculosis* in their surface, oligosaccharides, might have led to differences in the MBL levels associated with resistance or susceptibility against PTB [27, 44, 45]. Consequently, when geographic variation in pathogen polymorphism is superimposed on host genetic heterogeneity, considerable variation may occur in detectable allelic association [5]. These factors could explain our findings in the analysed Northern Spanish population.

## 6. Conclusion

The results obtained in our study show a significant higher prevalence of *MBL2* D allele and HYPD haplotype in controls than in PTB patients (6.7% versus 2.3%, *P* = 0.014, OR 0.33, and CI 95% 0.13–0.84). 

Although after statistical correction, the significance disappeared; this trend of the *P* values to significance could indicate a role of D allele and HYPD haplotype in conferring protection against PTB. For this reason, we cannot argue that *MBL2* D allele or HYPD haplotype would act as a factor of resistance to PTB, and susceptibility to this disease would probably be determined by other environmental and genetic factors, at least in our population [20, 46–50].

## Figures and Tables

**Table 1:** Allelic frequencies of *MBL2* structural variants and promoter polymorphisms in healthy controls and patients with pulmonary tuberculosis.

	Structural variants				Promoter polymorphisms					
	A	B	C	D	L	H	Y	X	P	Q
Controls										
(*n* = 441)	0.782	0.143	0.008	**0.067**	0.655	0.345	0.760	0.240	0.815	0.195
PTB Patients										
(*n* = 107)	0.836	0.131	0.009	**0.023**	0.678	0.322	0.776	0.224	0.757	0.243
*P* value^a,b^				**0.014**						
OR				**0.33**						
(95% CI)				**(0.13–0.84)**						

^a^Only D variant showed significant differences between the two groups. The rest of structural and promoter alleles did not show significant differences.

^b^The study had 74% power for detecting an odds ratio (OR) ≥ 2.

**Table 2:** Frequency of *MBL2* haplotypes in controls and PTB patients from Cantabria compared with other previously reported populations.

Haplotype	Population										
	Controls	PTB	CAN	ESK	DAN	JAP	KEN	MOZ	CHIR	MAP	WAR
	(*n* = 441)	(*n* = 107)	(*n* = 344)	(*n* = 72)	(*n* = 250)	(*n* = 218)	(*n* = 61)	(*n* = 154)	(*n* = 43)	(*n* = 25)	(*n* = 190)
HYPA	0.28	0.30	0.24	0.81	0.31	0.44	0.08	0.06	0.54	0.38	0.75
LYQA	0.23	0.23	0.22	0	0.19	0.16	0.25	0.27	0.01	0	0.01
LYPA	0.07	0.08	0.08	0.04	0.04	0.07	0.13	0.30	0.02	0.08	0.23
LXPA	0.21	0.23	0.19	0.03	0.26	0.11	0.24	0.13	0.01	0.04	0.01
LYPB	0.14	0.13	0.17	0.12	0.11	0.22	0.02	0	0.42	0.46	0
LYQC	0.01	0.01	0.03	0	0.03	0	0.24	0.24	0	0.04	0
HYPD	0.07	**0.02** ^ a^	0.07	0	0.06	0	0.04	0	0	0	0.003

H^b^	0.81	0.82	0.82	0.33	0.79	0.72	0.81	0.76	0.54	0.66	0.39

Controls, healthy population from Cantabria, PTB, patients with pulmonary tuberculosis from Cantabria, CAN, and Gran Canaria p; ESK, Eskimo; DAN, Danish; JAP, Japanese; KEN, Kenya; MOZ, Mozambique; CHIR, Chiriguano (South America); MAP, Mapuche (South America); WAR, Warlpiri (Australia) populations.

^a^ *P* value 0.014 (OR 0.33 and 95% CI 0.13–0.84): frequency of HYPD haplotype in PTB patients versus control subjects from Cantabria.

^b^Average heterozygosity of the seven alleles when they were considered together.

**Table 3:** Frequencies of complete diplotypes in Spanish PTB patients and controls.

*MBL2 *diplotypes*	PTB patients *n* (%)	Controls *n* (%)	*P* value	OR	(95% CI)
LYQA/HYPA	17 (15.9)	54 (12.24)	0.40	1.35	(0.75–2.45)
LYQA/LYPB	10 (9.3)	29 (6.58)	0.43	1.46	(0.69–3.11)
LYQA/LYPA	6 (5.6)	19 (4.31)	0.60	1.32	(0.51–3.39)
LYQA/LYQA	3 (2.8)	19 (4.31)	0.59	0.64	(0.19–2.20)
LYQA/HYPD	1 (0.9)	10 (2.27)	0.70	0.41	(0.05–3.21)
LYQA/LYQC	0 (0)	2 (0.45)	1.0	NA	NA
LYQC/HYPD	0 (0)	1 (0.23)	1.0	NA	NA
LXPA/HYPA	15 (14.0)	45 (10.20)	0.34	1.43	(0.77–2.69)
LXPA/LYQA	10 (9.3)	53 (12.02)	0.54	0.75	(0.37–1.54)
LXPA/LXPA	7 (6.5)	14 (3.17)	0.15	2.14	(0.84–5.43)
LXPA/LYPB	4 (3.7)	31 (7.03)	0.30	0.51	(0.18–1.49)
LXPA/HYPD	3 (2.8)	13 (2.95)	1.0	0.95	(0.27–3.39)
LXPA/LYPA	1 (0.9)	10 (2.27)	0.70	0.41	(0.05–3.21)
LXPA/LYQC	1 (0.9)	2 (0.45)	0.48	2.07	(0.19–23.05)
HYPA/HYPA	10 (9.3)	38 (8.62)	0.96	1.09	(0.53–2.27)
LYPB/HYPA	8 (7.5)	38 (8.62)	0.88	0.86	(0.39–1.89)
LYPB/LYPB	1 (0.9)	7 (1.59)	1.0	0.58	(0.07–4.81)
LYPB/LYQC	1 (0.9)	1 (0.23)	0.35	4.15	(0.26–66.90)
LYPB/HYPD	0 (0)	9 (2.04)	0.22	NA	NA
LYPA/HYPA	4 (3.7)	18 (4.08)	1.0	0.91	(1.30–2.75)
LYPA/LYPB	3 (2.8)	4 (0.91)	0.14	3.15	(0.69–14.30)
LYPA/LYPA	1 (0.9)	2 (0.45)	0.48	2.07	(0.19–23.05)
LYPA/LYQC	0 (0)	1 (0.23)	1.0	NA	NA
LYPA/HYPD	1 (0.9)	2 (0.45)	0.48	2.07	(0.19–23.05)
HYPD/HYPA	0 (0)	14 (3.17)	0.08	NA	NA
HYPD/HYPD	0 (0)	5 (1.13)	0.59	NA	NA

^∗^Frequencies of the rest of combined diplotypes (LYQC/HYPA, LYQC/LYQC) were 0 in both groups.

## References

[1] World Health Organization Global tuberculosis control: surveillance, planning, financing.

[2] Díez M, Huerta C, Moreno T (2002). Tuberculosis in Spain: epidemiological pattern and clinical practice. *International Journal of Tuberculosis and Lung Disease*.

[3] Pai M, Kalantri S, Dheda K (2006). New tools and emerging technologies for the diagnosis of tuberculosis: part I. Latent tuberculosis. *Expert Review of Molecular Diagnostics*.

[4] Pai M, Kalantri S, Dheda K (2006). New tools and emerging technologies for the diagnosis of tuberculosis: part II. Active tuberculosis and drug resistance. *Expert Review of Molecular Diagnostics*.

[5] Selvaraj P (2004). Host genetics and tuberculosis susceptibility. *Current Science*.

[6] Schurr E (2007). Is susceptibility to tuberculosis acquired or inherited?. *Journal of Internal Medicine*.

[7] Bellamy R (2006). Genome-wide approaches to identifying genetic factors in host susceptibility to tuberculosis. *Microbes and Infection*.

[8] Worthley DL, Bardy PG, Mullighan CG (2005). Mannose-binding lectin: biology and clinical implications. *Internal Medicine Journal*.

[9] Weis WI, Drickamer K, Hendrickson WA (1992). Structure of a C-type mannose-binding protein complexed with an oligosaccharide. *Nature*.

[10] Fraser IP, Koziel H, Ezekowitz RAB (1998). The serum mannose-binding protein and the macrophage mannose receptor are pattern recognition molecules that link innate and adaptive immunity. *Seminars in Immunology*.

[11] Thiel S, Vorup-Jensen T, Stover CM (1997). A second serine protease associated with mannan-binding lectin that activates complement. *Nature*.

[12] Neth O, Jack DL, Dodds AW, Holzel H, Klein NJ, Turner MW (2000). Mannose-binding lectin binds to a range of clinically relevant microorganisms and promotes complement deposition. *Infection and Immunity*.

[13] Eisen DP (2010). Mannose-binding lectin deficiency and respiratory tract infection. *Journal of Innate Immunity*.

[14] Sumiya M, Super M, Tabona P (1991). Molecular basis of opsonic defect in immunodeficient children. *The Lancet*.

[15] Madsen HO, Garred P, Thiel S (1995). Interplay between promoter and structural gene variants control basal serum level of mannan-binding protein. *Journal of Immunology*.

[16] Larsen F, Madsen HO, Sim RB, Koch C, Garred P (2004). Disease-associated mutations in human mannose-binding lectin compromise oligomerization and activity of the final protein. *The Journal of Biological Chemistry*.

[17] Garred P, Larsen F, Seyfarth J, Fujita R, Madsen HO (2006). Mannose-binding lectin and its genetic variants. *Genes and Immunity*.

[18] Casanova JL, Abel L (2004). Human mannose-binding lectin in immunity: friend, foe, or both?. *Journal of Experimental Medicine*.

[19] Singla N, Gupta D, Joshi A, Batra N, Singh J, Birbian N (2012). Association of mannose-binding lectin gene polymorphism with tuberculosis susceptibility and sputum conversion time. *International Journal of Immunogenetics*.

[20] Liu ZB, Zheng RJ, Xiao HP (2011). The correlation between polymorphisms of genes with susceptibility to tuberculosis and the clinical characteristics of tuberculosis in 459 Han patients. *Zhonghua Jie He He Hu Xi Za Zhi*.

[21] Solğun HA, Taştemir D, Aksaray N, Inan I, Demirhan O (2011). Polymorphisms in NRAMP1 and MBL2 genes and their relations with tuberculosis in Turkish children. *Tuberkuloz ve Toraks*.

[22] Denholm JT, McBryde ES, Eisen DP (2010). Mannose-binding lectin and susceptibility to tuberculosis: a meta-analysis. *Clinical and Experimental Immunology*.

[23] Dossou-Yovo OP, Lapoumeroulie C, Hauchecorne M (2009). Variants of the mannose-binding lectin gene in the benin population: heterozygosity for the p.G57E allele may confer a selective advantage. *Human Biology*.

[24] Alagarasu K, Selvaraj P, Swaminathan S, Raghavan S, Narendran G, Narayanan PR (2007). Mannose binding lectin gene variants and susceptibility to tuberculosis in HIV-1 infected patients of South India. *Tuberculosis*.

[25] Liu W, Zhang F, Xin ZT (2006). Sequence variations in the MBL gene and their relationship to pulmonary tuberculosis in the Chinese Han population. *International Journal of Tuberculosis and Lung Disease*.

[26] Selvaraj P, Jawahar MS, Rajeswari DN, Alagarasu K, Vidyarani M, Narayanan PR (2006). Role of mannose binding lectin gene variants on its protein levels and macrophage phagocytosis with live *Mycobacterium tuberculosis* in pulmonary tuberculosis. *FEMS Immunology and Medical Microbiology*.

[27] García-Laorden MI, Pena MJ, Caminero JA (2006). Influence of mannose-binding lectin on HIV infection and tuberculosis in a Western-European population. *Molecular Immunology*.

[28] El Sahly HM, Reich RA, Dou SJ, Musser JM, Graviss EA (2004). The effect of mannose binding lectin gene polymorphisms on susceptibility to tuberculosis in different ethnic groups. *Scandinavian Journal of Infectious Diseases*.

[29] Søborg C, Madsen HO, Andersen AB, Lillebaek T, Kok-Jensen A, Garred P (2003). Mannose-binding lectin polymorphisms in clinical tuberculosis. *Journal of Infectious Diseases*.

[30] García-Laorden MI, Manzanedo A, Figuerola A, Sánchez-García F, Rodríguez-Gallego C (2001). Mannose-binding lectin polymorphisms in a Canary Islands (Spain) population. *Genes and Immunity*.

[31] Özbaş-Gerçeker F, Tezcan I, Berkel AI (2003). The effect of mannose-binding protein gene polymorphisms in recurrent respiratory system infections in children and lung tuberculosis. *Turkish Journal of Pediatrics*.

[32] Mombo LE, Lu CY, Ossari S (2003). Mannose-binding lectin alleles in sub-Saharan Africans and relation with susceptibility to infections. *Genes and Immunity*.

[33] Hill AVS (1998). The immunogenetics of human infectious diseases. *Annual Review of Immunology*.

[34] Garred P, Richter C, Andersen AB (1997). Mannan-binding lectin in the sub-saharan HIV and tuberculosis epidemics. *Scandinavian Journal of Immunology*.

[35] Hoal-van Helden EG, Epstein J, Victor TC (1999). Mannose-binding protein B allele confers protection against tuberculous meningitis. *Pediatric Research*.

[36] Selvaraj P, Narayanan PR, Reetha AM (1999). Association of functional mutant homozygotes of the mannose binding protein gene with susceptibility to pulmonary tuberculosis in India. *Tubercle and Lung Disease*.

[37] Delgado JC, Baena A, Thim S, Goldfeld AE (2002). Ethnic-specific genetic associations with pulmonary tuberculosis. *Journal of Infectious Diseases*.

[38] Flores C, Maca-Meyer N, Pérez JA, González AM, Larruga JM, Cabrera VM (2003). A predominant European ancestry of paternal lineages from Canary Islanders. *Annals of Human Genetics*.

[39] Pinto F, González AM, Hernández M, Larruga JM, Cabrera VM (1996). Genetic relationship between the Canary Islanders and their African and Spanish ancestors inferred from mitochondrial DNA sequences. *Annals of Human Genetics*.

[40] Pinto FM, González AM, Hernández M, Larruga JM, Cabrera VM (1996). Sub-Saharan influence on the Canary Islands population deduced from G6PD gene sequence analysis. *Human Biology*.

[41] Esteban E, Dugoujon JM, Guitard E (1998). Genetic diversity in Northern Spain (Basque Country and Cantabria): GM and KM variation related to demographic histories. *European Journal of Human Genetics*.

[42] Sánchez-Velasco P, Escribano de Diego J, Paz-Miguel JE, Ocejo-Vinyals G, Leyva-Cobián F (1999). HLA-DR, DQ nucleotide sequence polymorphisms in the Pasiegos (Pas valleys, Northern Spain) and comparison of the allelic and haplotypic frequencies with those of other European populations. *Tissue Antigens*.

[43] Sánchez-Velasco P, Gómez-Casado E, Martínez-Laso J (2003). HLA alleles in isolated populations from north Spain: origin of the basques and the ancient Iberians. *Tissue Antigens*.

[44] Kato-Maeda M, Bifani PJ, Kreiswirth BN, Small PM (2001). The nature and consequence of genetic variability within *Mycobacterium tuberculosis*. *Journal of Clinical Investigation*.

[45] López B, Aguilar D, Orozco H (2003). A marked difference in pathogenesis and immune response induced by different *Mycobacterium tuberculosis* genotypes. *Clinical and Experimental Immunology*.

[46] Keicho N, Hijikata M, Sakurada S (2011). Human genetic susceptibility to tuberculosis. *Nihon Rinsho*.

[47] Wilkinson RJ (2012). Human genetic susceptibility to tuberculosis: time for a bottom-up approach?. *Journal of Infectious Diseases*.

[48] Rowell JL, Dowling NF, Yu W, Yesupriva A, Zhang L, Gwinn M (2012). Trends in population-based studies of human genetics in infectious diseases. *PloS ONE*.

[49] Qidwai T, Jamal F, Khan MY (2012). DNA sequence variation and regulation of genes involved in pathogenesis of pulmonary tuberculosis. *Scandinavian Journal of Immunology*.

[50] Azad AK, Sadee W, Schlesinger LS (2012). Innate immune gene polymorphisms in tuberculosis. *Infection and Immunity*.

